# Detection and Identification of Various Microplastics in Different Orthodontic Adhesives

**DOI:** 10.7759/cureus.55221

**Published:** 2024-02-29

**Authors:** Anjusha Divakar, Shantha K Sundari, Sivakamavalli Jeyachandran

**Affiliations:** 1 Orthodontics and Dentofacial Orthopaedics, Saveetha Dental College and Hospitals, Saveetha Institute of Medical and Technical Sciences, Saveetha University, Chennai, IND

**Keywords:** resin-based composites, fourier-transform infrared spectroscopy, scanning electron microscope, microplastics, orthodontic adhesives

## Abstract

Background

Microplastics are acknowledged as significant environmental contaminants. The clinical use of dental materials, particularly adhesives containing plastic polymers, can give rise to the production of plastic micro- and nanoparticles, which subsequently find their way into the environment. The aim of the study was to detect different microplastics and identify them in various orthodontic adhesives.

Materials and methods

Four different light cure orthodontic adhesives, including Transbond XT (3M Unitek, Monrovia, CA), Ormco Enlight (Ormco, Orange, CA), Orthofix SPA (Orthofix, Verona, Italy), and Aqualine LC (Tomy International Inc, Tokyo, Japan), were collected and placed in separate Eppendorf tubes. Microplastics present in each adhesive were identified using scanning electron microscopy. Subsequently, each specimen was suspended in hydrogen peroxide, placed within a shaking incubator, and analyzed using Fourier transform infrared spectroscopy (FTIR) to identify the type of polymer.

Results

The scanning electron microscope shows the surface morphology and the most predominant types of microplastics identified were fibers, fragments, and pellets. FTIR results showed the presence of several major functional groups, including hydroxyl, amine, ester, fluoro, and halo groups.

Conclusion

When contrasted with the quantity of microplastic waste generated by other sectors like the textile, cosmetic, and fishing industries, the microparticulate waste stemming from dental adhesives has a minimal effect on environmental deterioration. Strategies for addressing this concern should give precedence to reducing the use of these materials and adopting effective recovery methods, which could potentially involve recycling processes.

## Introduction

Dental adhesives play an indispensable role in modern dentistry, serving as a crucial component in the bonding of restorative materials, such as dental composites and prosthetic devices, to natural teeth. These adhesives are designed for durability and biocompatibility to ensure the long-term success of dental procedures. Dental adhesives undergo thorough testing to ensure they are biocompatible and meet high-quality and safety standards such as the United States Pharmacopeia (USP) VI standardization and International Organization for Standardization (ISO) 10993 certification. These adhesives are considered to be safe for human use and are of medical-grade quality. These adhesive systems are carefully formulated to achieve specific bonding properties and performance characteristics. A resin-based matrix bisphenol A-glycidyl methacrylate (BISGMA), urethane dimethacrylate (UDMA), or semi-crystalline polyceram (PEX), and an inorganic filler like silicon dioxide (silica) make up an adhesive system. The resin matrix provides the adhesive's structural integrity and bonding strength. Fillers enhance the adhesive's mechanical properties, such as stiffness and wear resistance, contributing to the overall strength and longevity of the bond. Initiators and accelerators are chemicals that facilitate the polymerization process of the resin matrix [1,2].

Microplastics, defined as tiny plastic particles with dimensions less than 5 millimeters, have become a global environmental concern due to their widespread presence in various ecosystems, including aquatic environments [3], soil, and even the air we breathe [4-6]. These tiny plastic particles come from a variety of origins, such as the degradation of larger plastic objects, the wear and tear of synthetic fabrics, and the intentional incorporation of microplastics in personal care and industrial goods [7-9]. While much attention has been directed toward understanding their impact on the natural environment and human health, there remains a lesser-explored facet of microplastics, i.e., their presence in dental adhesives. However, the potential inclusion of microplastics within dental adhesives raises concerns about their unintended introduction into the oral environment during routine dental procedures as well as their subsequent impact on patient health [10]. To date, limited research has been conducted to investigate the presence and characteristics of microplastics in dental adhesives, despite the growing interest in understanding their occurrence in various consumer products. Given the potential for exposure and the lack of comprehensive data in this specific context, there is a compelling need to undertake systematic studies aimed at isolating and characterizing microplastics within dental adhesives. Such research endeavors are essential not only to assess potential health risks but also to guide the dental industry toward more sustainable and environmentally responsible product formulations. In this research, we offer an extensive examination of the process of isolating and analyzing microplastics found in four distinct dental adhesives. This study will give new insights into bioadhesives and novel biomaterials production in medical appliances; additionally, such materials would reduce the risk of microplastic accumulation. Through a series of physicochemical identification techniques, such as Fourier transform infrared spectroscopy (FTIR) and scanning electron microscope (SEM), we aim to elucidate the presence, types, sizes, and potential sources of microplastics within dental adhesive formulations. Our research aims to illuminate an aspect of microplastic pollution that has not been explored before.

## Materials and methods

Experimental materials and microplastic extraction

**Figure 1:**
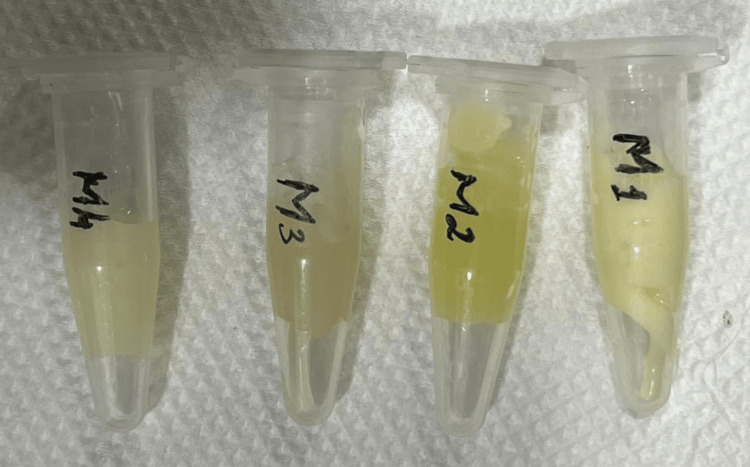
Different adhesives in Eppendorf vials.

Four different light cure orthodontic adhesives were used for the isolation and characterization of microplastics: M1 - Enlight (Ormco, Orange, CA); M2 - Transbond XT (3M Unitek, Monrovia, CA); M3 - Aqualine LC (Tomy International Inc, Tokyo, Japan); and M4 - Orthofix SPA (Orthofix, Verona, Italy). To characterize microplastics from commonly used sea salts, hydrogen peroxide was introduced to salts from various commercial brands. After shaking in an incubator, filtered distilled water was added to fully dissolve any clumped salt. The resulting mixture was promptly subjected to vacuum filtration using a vacuum filtration apparatus (YUXun YX, China) and confirmed the presence of microplastics [11,12]. This method has been employed for microplastic isolation from marine sediments, sea salts, and various other samples but not in dental samples, therefore the methodology has been slightly modified for dental adhesive samples as the digestion of adhesive samples takes a longer period of time. The adhesives were taken in different Eppendorf vials and 30% H2O2 was mixed and transferred to glass vials to avoid cross-contamination of plastics in the sample, and then the glass vials were placed in a water bath for one hour at 60°C (Figure 1) [12].

After 12 hours, the vials containing orthodontic adhesives were placed in 100 mL of saturated NaCl solution. The adhesives soaked in NaCl solution were mixed and covered with aluminum foil, standing for 12 hours for the density separation process. The supernatant of adhesives mixed with 1N NaCl was filtered using a filter membrane (Merck, Rahway, NJ) with a pore size of 0.45 µm x 47 mm diameter [12].

The filter was first rinsed with pure distilled water to remove NaCl residues and then placed in a glass petri dish to dry. Subsequently, approximately 15 mL of hydrogen peroxide was added to the sample to eliminate organic matter. The sample was then placed in a shaking incubator (RivoTEK, New Delhi, India) set at 45°C with an agitation rate of 75 rpm for 24 hours. After incubation, the mixture was allowed to reach room temperature and left undisturbed for 48 hours. To ensure complete dissolution of any clumped salt, 850 mL of previously filtered distilled water was added. The resulting liquid was then subjected to vacuum filtration using cellulose nitrate filter paper with 5-millimeter pores and a 47-millimeter diameter. The filter papers were changed at designated time intervals and left to air dry at room temperature to facilitate the examination and characterization of microplastics [12].

Fourier transform infrared spectroscopy (FTIR)

**Figure 2:**
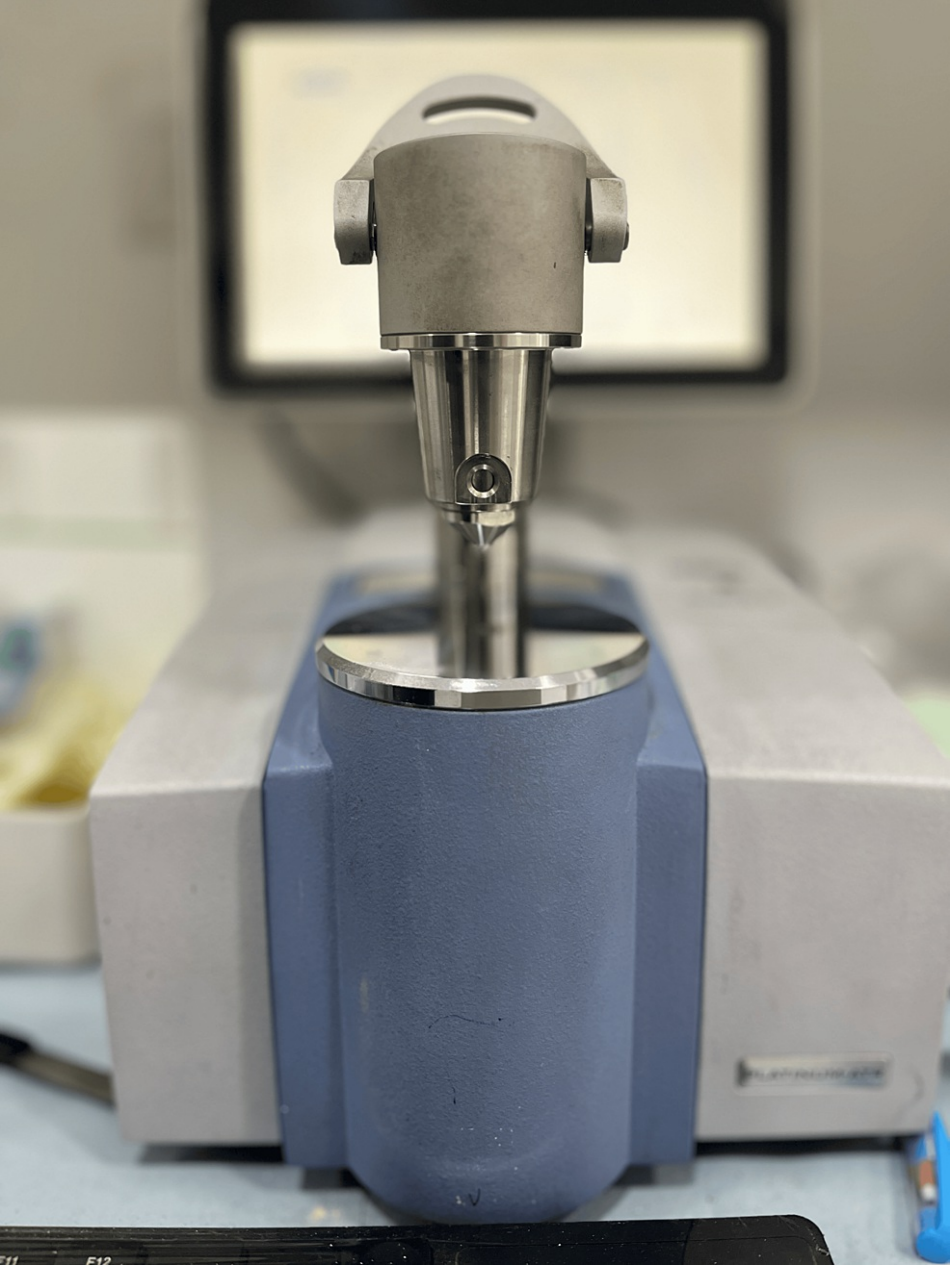
Fourier transform infrared spectroscopy (ALPHA II Compact FTIR Spectrometer, Bruker, Billerica, MA).

Microscopic examination was used to choose specific portions of microplastics (MPs), which were subsequently subjected to FTIR spectroscopy (ALPHA II Compact FTIR Spectrometer, Bruker, Billerica, MA) analysis in transmittance mode; the spectral range was configured from 4000 to 400 cm⁻¹, and the resulting data were compared with a standard library (Hummel Polymer and Additives) for polymer type identification (Figure 2). Mid-infrared and tungsten-halogen source lamps were used as a light source. Spectra with a match quality index exceeding 0.7 were considered, while matches below 0.6 were largely excluded from consideration as those components in that spectral range do not come under microplastic polymers [13].

Scanning electron microscopy (SEM)

SEM allowed for the visualization of the physical structure and surface characteristics of the MPs at high magnification, providing insights into their shape, size, and surface topography. Sample preparation for SEM imaging entails the collection, cleaning, and placement of microplastics onto an appropriate substrate. To enhance conductivity and mitigate the occurrence of charging artifacts, a thin conductive layer was applied to the microplastics. The SEM chamber serves as the location where the sample is housed, enabling the focused electron beam to interact with the surface of the microplastics. The instrument settings should be optimized to ensure accurate imaging and analysis.

## Results

**Figure 3:**
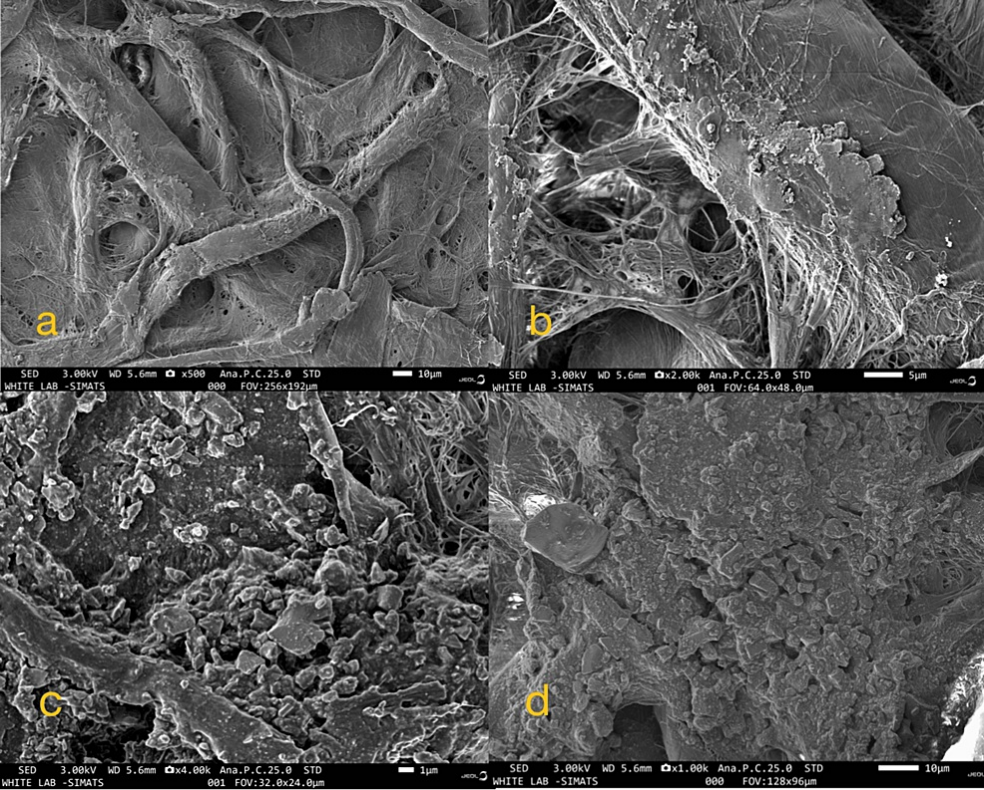
(a) SEM image of M1, (b) SEM image of M2, (c) SEM image of M3, and (d) SEM image of M4. M1: Fibrous MPs. M2: Thin fibers and fine small granules. M3: Deformed unidentified structures with certain hexagonal-shaped MPs. M4: Clustered MP shapes. M1 - Ormco Enlight; M2 - Transbond XT; M3 - Aqualine LC; M4 - Orthofix SPA. SEM: scanning electron microscope; MP: microplastic.

In our investigation, we utilized a technique involving hydrogen peroxide to disintegrate organic components in the samples, thereby enabling the extraction of MPs, approximately 98%, leading to their removal from dental adhesives. We conducted a comprehensive investigation into the presence of MPs in commercial dental adhesive products, encompassing four different brands from diverse origins. Due to the varying density of these particles, we regularly replaced the filter paper when we observed color intensification. The filter paper captured the majority of the particles, and these were predominantly transparent and devoid of color. However, it is worth noting that some of the particles exhibited a variety of hues, including black, red, white, and blue. Notably, we also identified dusty particles with uncertain origins in certain instances. Across all samples, the average number of MPs per pack of dental adhesive was found to be less than 150 particles. The greatest concentration of MPs was identified in M3-Aqualine LC, whereas M1-Ormco Enlight exhibited the lowest level. Interestingly, we found no noteworthy variances in the prevalence of MPs among the four distinct samples. Our analysis revealed a diverse composition of MPs, encompassing fibers, fragments, pellets, and dusty materials of unidentified origin. Concurrently, the most prevalent types of MPs identified were fibers, fragments, and pellets (as depicted in Figure 3). M1-Ormco Enlight has fibrous MPs, M2-Transbond XT shows thin fibers and fine small granules, M3-Aqualine LC shows deformed unidentified structures with certain hexagonal shaped MPs, and M4-Orthofix SPA depicts clustered MPs shape. Additionally, materials with uncertain origins were detected in all four dental adhesive samples. In our research, we documented a wide range of microplastic sizes, with the smallest particles measuring as small as 2.6 millimeters and the largest ones reaching up to 3.3 millimeters in size. This variation in microplastic sizes underscores the diversity and complexity of these particles within the samples we examined. Moreover, we utilized SEM to visually validate the existence of MPs, as illustrated in Figure 3.

According to the FTIR analysis of MPs, the C=O functional group was found at the wavelength of 1550-1700 cm-1 in M2, and in M4 samples, the CH and CH2 group was identified at the wavelength of 605-800 cm-1 and 1019-1300 cm-1 in all four samples, respectively. The N-H group was found at 3330 cm-1 in M1 and M3. The C-C group was found at the wavelength of 1019-1030 cm-1 in all samples. The C-H functional group was identified at a wavelength of 2896-2923 cm-1 in all four samples.

**Figure 4:**
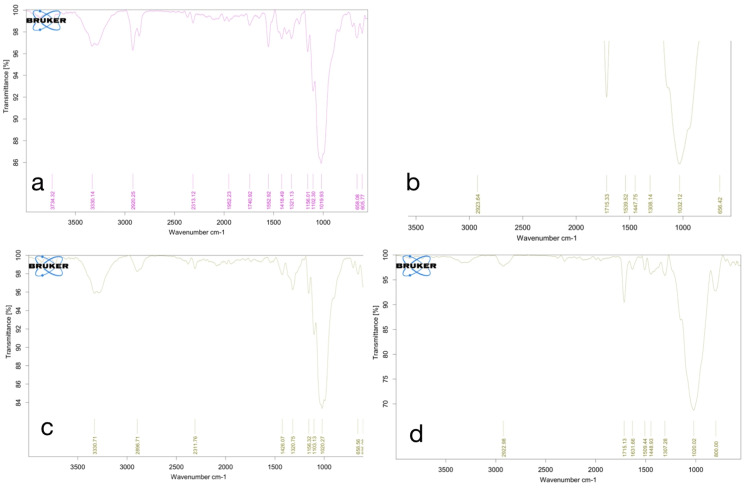
(a) FTIR spectra of M1, (b) FTIR spectra of M2, (c) FTIR spectra of M3, and (d) FTIR spectra of M4. M1 - Ormco Enlight; M2 - Transbond XT; M3 - Aqualine LC; M4 - Orthofix SPA. FTIR: Fourier transform infrared spectroscopy.

According to Song et al., FTIR analysis is a method for verifying the presence of MPs based on their polymer chemical components [13]. In our investigation, a set of prevalent microplastic types, including fragments, fibers, and pellets, sourced from widely recognized and commercially available dental adhesives, underwent FTIR analysis to identify their polymer composition. We considered a match of approximately 70-75% as an acceptable indicator of polymer identity, following the criteria proposed by Woodall et al. in 2014 [14]. The FTIR histogram in Figure 4 displayed the identification of four predominant polymer types: polyamide (PA), polypropylene (PP), polystyrene (PS), and cellophane (CP), with a remarkable 93% matching identity. Among all the adhesive samples, the maximum proportion of MPs followed this order: polyamide > polypropylene > polystyrene > cellophane. Additionally, a minimal amount of MPs such as polyethylene terephthalate (PET) and polyethylene was detected in our study.

## Discussion

Dental adhesives are complex combinations of various components. A deep understanding of these constituents is essential for gaining insights into how adhesives behave in both research settings and clinical practice. This comprehensive understanding also offers valuable insights into the appropriate clinical application of adhesives. Each ingredient has a degree of influence on factors such as bond strength, bonding efficiency, bonding durability, shelf life, and biocompatibility within the adhesive system [15]. Conversely, a carefully planned formulation is the cornerstone of achieving long-term success in clinical applications. In recent times, MPs have become a worldwide concern and are widely acknowledged as being present everywhere [16]. Consequently, the potential danger of animals and humans being exposed to MPs is on the rise. In a study by Lucy et al., the diverse range of polymer types identified indicates that the complex accumulation and settling of microfibers in the deep sea result from various domestic and industrial origins [17].

According to a study done in 2021 by Sivagami et al., the findings revealed that various types of MPs were present in salts from 10 different Indian commercial brands, originating from diverse sources. These results provide evidence of human influence on the progressive buildup of MPs in ocean environments [12]. In a different study by Madhumitha et al. done in 2022, where researchers extracted, identified, and assessed the risk of microplastics in different kinds of toothpaste available in the Indian market, they found microplastics with variations in size categories, dispersion, and polymer compositions. The presence of microplastic particles in toothpaste ranged from 0.2% to 0.9% by weight, and the predominant forms were fibers and fragments. Long-term use of microbead-containing toothpaste can lead to tooth enamel and dentin abrasion. Microbeads can become lodged in the gingival sulcus, causing gingivitis and periodontitis [15]. Exposure to microplastics can lead to minimal uptake, with absorption rates typically remaining below 1% [18]. When compared to the amount of plastic microparticle waste from other industries, microparticulate waste from dental adhesives has a negligible impact (0.1/100) when compared to environmental degradation [19]. Moreover, this study carries importance as the health sector is morally bound to uphold sustainability, keeping in mind the possibility of adverse effects and, most importantly, making proactive efforts to avert harm.

Microplastics from industrial sources originate from the degradation of larger plastic materials used in manufacturing processes. Conversely, microplastics from dental adhesives result from the breakdown of acrylic-based materials commonly used in dental treatments. Industrial microplastics vary widely in size, shape, and composition, posing environmental risks such as water pollution and wildlife ingestion. Dental microplastics are smaller and uniform, often consisting of acrylic polymers specific to dental materials, potentially leading to oral health concerns. Understanding these differences is crucial for developing targeted mitigation strategies to minimize the impacts of microplastics on both the environment and human health.

This study has created a tool by establishing and confirming a technique to separate and identify microplastics in dental adhesives. By comparing the data from all samples to information on related materials stored in a database, it becomes possible to detect microparticle waste downstream of dental offices and laboratories. The characterization of dental adhesive-derived microparticles advances our fundamental understanding in this area and motivates recommendations for additional research. It is required to validate and expand upon this initial model. Furthermore, a deeper exploration of the health impacts of microplastics within the human body is essential.

Limitations

It is important to mention that we did not perform FTIR analysis on the unidentified dust particles. Information about the mass of MPs is not accessible with this method. One of the major drawbacks of this technique is that very small particles <10 μm cannot be analyzed as long infrared (IR) wavelengths (2.5 μm to 20 μm) limit the lateral resolution of IR microscopy to several millimeters as it can interact with similar-sized particles only. Micro FTIR can be used to identify some of the acrylic polymers, such as polyacrylic acid, polymethyl methacrylate, and poly butyl acrylate.

In a study focused on the presence of various MPs in dental materials, it is essential to underscore the need for quantitative analysis and comparison between different types of adhesives. While the study may provide valuable insights into the diversity and prevalence of MPs, it lacks quantitative assessments that could elucidate the extent of MP contamination and its potential implications for human health. Thus, future research efforts should prioritize quantitative analyses to better understand the distribution, abundance, and potential health risks associated with MPs in aquatic ecosystems. By addressing these gaps in knowledge, researchers can contribute to more informed decision-making and policy development aimed at mitigating the adverse effects of MP pollution on both environmental and human health.

## Conclusions

The study found microplastics ranging from 2.6 to 3.3 millimeters in size in various forms, such as fibers, fragments, and pellets, in orthodontic adhesives. SEM analysis revealed their surface features and FTIR analysis identified key functional groups, including hydroxyl, amine, ester, fluoro, and halo groups.
